# Sexually Dimorphic Associations between Maternal Factors and Human Milk Hormonal Concentrations

**DOI:** 10.3390/nu12010152

**Published:** 2020-01-06

**Authors:** Laura Galante, Hanna Lagström, Mark H. Vickers, Clare M. Reynolds, Samuli Rautava, Amber M. Milan, David Cameron-Smith, Shikha Pundir

**Affiliations:** 1 The Liggins Institute, University of Auckland, Auckland 1023, New Zealand; l.galante@auckland.ac.nz (L.G.); c.reynolds@auckland.ac.nz (C.M.R.); a.milan@auckland.ac.nz (A.M.M.); d.cameron-smith@auckland.ac.nz (D.C.-S.); s.pundir@auckland.ac.nz (S.P.); 2 Department of Public Health, University of Turku and Turku University Hospital, 20014 Turku, Finland; hanna.lagstrom@utu.fi; 3 Department of Pediatrics, University of Turku and Turku University Hospital, 20521 Turku, Finland; samrau@utu.fi; 4 Singapore Institute for Clinical Sciences, Agency for Science, Technology and Research, Singapore 117609, Singapore

**Keywords:** Breastmilk composition, IGF-1, Adiponectin, Leptin, cGP, Protein, Sex-specific milk composition, Gestational diabetes, Body mass index

## Abstract

While human milk composition is characterised by marked dynamicity, we are far from having a clear picture of what factors drive this variation. Hormones in human milk are known to vary according to specific maternal phenotypes, but limited evidence shows the infant also has a role in determining milk composition. The present study aimed to investigate the interplay between maternal and infant characteristics in relation to human milk hormonal profile. In total, 501 human milk samples from mothers recruited in the Finnish STEPS cohort study (Steps to the healthy development) were analysed. Pre-pregnancy and pregnancy maternal data, socioeconomic status and infant characteristics at birth were collated. Leptin, adiponectin, insulin-like growth factor-1 and cyclic Glycine-Proline in milk were measured. Multivariate analysis of variance (MANOVA) and linear regression were utilised for statistical analysis. Sex-specific interactions with maternal factors were observed, as the infant sex mediated associations between gestational diabetes and milk adiponectin (*p* = 0.031), birth-mode and total protein (*p* = 0.003), maternal education and insulin-like growth factor-1: cyclic Glycine-Proline ratio (*p* = 0.035). Our results suggest that changes in human milk composition are associated with interactions between maternal and infant characteristics and pathophysiological factors. Future work should expand on these findings and further explore the link between hormonal profiles in human milk and infant outcomes.

## 1. Introduction

Biologically active compounds in human milk (HM) include different classes of hormones, such as growth [1] and satiety factors [2], that play important roles in human physiology and metabolism mediating energy intake [3] and somatic growth [4]. Extensive evidence from animal models has shown that these compounds are likely to be active in HM and, once ingested by the infant, exert a range of physiological functions [5]. Changes in HM nutritional and hormonal composition have been well documented throughout the sequential stages of lactation [6,7], per feed [8], per day [9], across mothers with different phenotypic characteristics [10,11], socioeconomic status [12] and infants of different sex [13]. Limited studies to date [11,14,15,16,17] have reported alterations in the HM bioactive, amino and fatty acid profiles of women with gestational diabetes mellitus (GDM) or elevated body mass index (BMI), suggesting that HM may have different nutritional and hormonal composition associated with individual maternal characteristics. This also suggests that certain concentrations might be more desirable than others in relation to their effects on infant growth [18], as it is well established that nutritional and hormonal exposures during the first 1000 days of life affect infant growth and metabolic health in the long term [19].

Hormones mediating metabolic function and growth are of particular importance in HM, as they influence a range of infant growth and developmental outcomes. Insulin-like growth factor 1 (IGF-1) is a key mediator of growth hormone action and the most important growth factor in the infant [20]. However, despite its importance in infant growth and regulation of fat accrual during childhood [21], only limited and outdated information is available around its role in HM and its impact on infant growth and development. In experimental animal models, cyclic Glycine-Proline (cGP), a metabolite of IGF-1 derived from cleavage of the N-terminal of IGF-1, has been shown to play a role in the regulation of IGF-1 bioavailability, with the ratio between the two compounds indicating the bioactivity of IGF-1 [22]. The presence and potential role of cGP in HM has not been investigated. Adipokines also constitute an important group of HM bioactives [23]. Amongst these, leptin is known to be involved in appetite and energy balance regulation [24] and to be altered in subjects with elevated BMI [25]. Other adipokines, such as adiponectin, are involved in the regulation of inflammatory processes and insulin sensitivity [26]. In experimental animal models, the presence of adipokines in HM has been linked to postnatal programming and may impact later metabolic health outcomes in the infant [5,27], including the development of obesity and metabolic syndrome later in life [28]. Nonetheless, with the exception of leptin, the concentration of these hormones in HM and the interplay between infant-maternal characteristics and HM composition remains poorly defined.

As both maternal and infant characteristics, particularly maternal metabolic status before and during pregnancy and infant sex, appear to be associated with HM composition, we hypothesised that interactions between the two influence HM composition, conferring a specific profile to each milk sample. Thus, we investigated the contribution of maternal factors before and during pregnancy and infant characteristics at birth with the hormonal composition of mature HM.

## 2. Materials and Methods

The present study is based on data and HM samples from 501 mothers and 507 children participating in a Finnish longitudinal cohort, Steps to healthy development of Children (the STEPS Study), which has previously been described in detail [29]. Briefly, all Finnish- and Swedish-speaking mothers who delivered a living child between 1 January 2008 and 31 April 2010 in the Hospital District of Southwest Finland formed the cohort population (in total 9811 mothers and their 9936 children). Altogether, 1797 mothers (18.3% of the total cohort) with 1827 neonates (including 30 pairs of twins) volunteered as participants for the intensive follow-up group of the STEPS study. Written informed consent was obtained from the participants. The study protocol was approved by the Ethics Committee of the Hospital District of South West Finland in February 2007. HM samples were collected approximately 2.6 ± 0.4 months after birth from participants who consented to provide HM. These accounted for 45% of the total sample size of the study (811/1797). In total, 310 samples were further excluded from the present study due to the lack of consent from mothers for secondary analysis on the samples, or insufficient sample volume. HM samples were collected as previously described [30] by manual expression in the morning, between 10 am and 12 pm. Before emptying the content of one breast in the provided container, mothers manually expressed the first few drops of foremilk to waste.

Self-reported height and weight before pregnancy and minimum and maximum weight during pregnancy were collected at recruitment and used to determine pre-pregnancy BMI and pregnancy weight gain. Mothers were then grouped into four categories according to their pre-pregnancy BMI (underweight < 18.5 kg/m^2^, normal weight 18.5–24.9 kg/m^2^, overweight 25–29.9 kg/m^2^, obese >29.9 kg/m^2^). Information regarding GDM (diagnosis based on the ICD-10-CM Codes O24), infant sex, birth weight and length were obtained from the Longitudinal Census Files. Birth weight Z scores were calculated using the published references specific to the Finnish population [31]. Socioeconomic information (maternal basic education, family income and main occupation of mother during pregnancy) were collected through prenatal questionnaires [29]. Information on feeding practices were collected through follow-up diaries completed by mothers [29]. Exclusive breastfeeding was defined as the infant not receiving anything other than HM, with the exception of water, supplements or medicines. Total breastfeeding was defined as the infant receiving HM and any other liquid or food.

Leptin and adiponectin analyses were performed using a commercially available enzyme-linked immunosorbent assay (ELISA) kit (human sensitive Leptin ELISA and human Adiponectin ELISA, Mediagnost, Germany). 150 µl of sample was centrifuged at 3000× *g* for 15 min and the skim milk under the fat layer was used for the assay. For leptin analysis, skim milk samples were diluted 1:20 and the assay performed as per manufacturer’s instructions. For adiponectin, skim milk samples were diluted 1:2 and the assay was performed as per manufacturer’s instructions. 

IGF-1 was analysed using a commercially available ELISA kit (human IGF-1 ELISA, Mediagnost, Germany). Skim HM samples were collected following centrifugation at 3000× *g* for 15 min and diluted 1:2 with acidified dilution buffer. After dilution, samples were centrifuged to precipitate the binding proteins and then analysed as per manufacturer’s instructions.

cGP was analysed through liquid chromatography tandem mass spectrometry (LC-MS), utilising a protocol developed and optimised by our laboratory on rat milk and published previously [32]. The protocol was adapted for analysis of HM. Briefly, 35 µL of cGP-d_2_ internal standard (175 mg/mL) was added to each sample. Samples were then passed through phospholipid removal cartridges (Phree phospholipid removal solutions, Phenomenex, Torrance, CA, USA) by performing an extraction with 1% (1:99 v/v) formic acid in acetonitrile. Following extraction, samples were vacuum-dried for 6 h and then reconstituted in a water-methanol solution (90:10 v/v) and injected into the machine. The mass spectrometer used a Surveyor MS pump and auto sampler followed by an Ion Max APCI source on a Finnigan TSQ Quantum Ultra AM triple quadruple mass spectrometer, all controlled by FinniganX caliber software (Thermo Electron Corporation, San Jose, CA, USA.). The column used was Kinetex^®^ 1.7 µL EVO C18 100 Å measuring 150 × 2.1 mm (Phenomenex, Torrance, CA, USA). The mobile phase was constituted by methanol-water gradient set at 95:5 (v/v) at a flow of 250 μL/min. Chromatography was performed at 40 °C. Argon was used as the collision gas at 1.2 mTorr. The concentration of cGP in samples was calculated from a standard curve generated for each assay (0.14 ng/mL–265.12 ng/mL). Inter- and intra assay variability was <10%.

HM total protein was quantified in order to normalise the hormone concentrations but also to investigate the associations with maternal-infant factors. The detection of total protein in the samples was carried out by infrared spectrometry using the Direct Detect^®^ technology (Merck, Germany). Following dilution in 1:5 with milli-Q water, 2 µL of HM samples were transferred to the Direct Detect^®^ Assay-free cards (Merck, Germany) and read with the Direct Detect^®^ spectrometer as per the manufacturer’s instructions. 

The adiponectin ELISA has previously been reported for use with HM [33]. Leptin ELISAs were used with antibodies previously validated for use with HM [34]. For all ELISAs, HM samples were tested for linearity (parallel displacement to the standard curve for diluted samples) and suitability of dilution/extraction method. The intra- and inter-assay coefficients of variation respectively for the ELISAs (QCs supplied) were adiponectin (5%, 6%), IGF-1 (3%, 9%) and leptin (4%, 8%). Samples were randomised for all assays.

The power provided by the sample size of the population for linear regression and multivariate analysis of variance (MANOVA) was calculated with G*Power 3.1.9.2 as greater than 90% at the 5% significance level for the detection of 10% difference across groups for all the measured main effects. HM concentration of adipokines, IGF-1 and cGP were reported as ng or mg of the total protein concentration per ml in each sample. This was done to allow a better alignment of the results across samples and across studies, by introducing a reference biomarker (protein) that is commonly used for this purpose. Normal distribution of the data was verified through the Shapiro-Wilk test. As concentrations of bioactive components were skewed, they were normalised by log10-transformation. Relationship between pairs of compounds were investigated with bivariate analysis. Simple and multiple linear regression was used to investigate relationship between bioactives concentrations in HM and scale variables such as maternal age, infant nutrition (exclusive breastfeeding, partial breastfeeding, introduction of solid foods), maternal weight gain during pregnancy, weight before pregnancy and infant gestational age, birthweight and birth length. To investigate possible predictors of HM composition amongst categorical values, we used MANOVA and tested the effect of single factors or pairs of factors on HM concentrations of all four bioactive compounds and on the ratio between IGF-1 and cGP [22]. Because some mothers did not exclusively breastfeed their infants at the time of sample collection, we used the duration of exclusive breastfeeding as a correcting factor in our analysis. Similarly, the introduction of solid foods for some infants in the cohort occurred before the milk sample was collected, and for this reason, we investigated the association between HM composition and introduction of solids. As we were specifically looking at sex-specific effects, each factor was paired with infant sex when fitting the relative MANOVA model. Other variables that were tested included maternal pre-pregnancy BMI, GDM, maternal basic education and main occupation during pregnancy, depression, birth-mode and multiple-single pregnancy. When multiple groups were compared, a Bonferroni correction was applied. All statistical analyses were performed using IBM SPSS (version 25) and the graphs generated by using GraphPad Prism 7 and RStudio.

## 3. Results

### 3.1. Population Characteristics

The main demographics of the study population are detailed in Table 1. Over 50% of the population presented with a normal BMI and less than 10% developed GDM (% of GDM mothers within the different BMI classes = 5.3% underweight, 4.1% normal weight, 17.6% overweight, 25.5% obese). A majority of mothers had a secondary school qualification and spent most of their pregnancy working full-time. Nearly half of the population (49%) reported exclusive breastfeeding for three months or less while the remainder (51%) reported exclusive breastfeeding for up to six and a half months. Overall, 80% of mothers reported a total breastfeeding (exclusive and partial) duration above 6 months. The introduction of solid foods occurred before 4 months of age for 42.2% of the population and after 4 months for the remaining infants (57.8%). 

### 3.2. HM Samples

**Figure 1:**
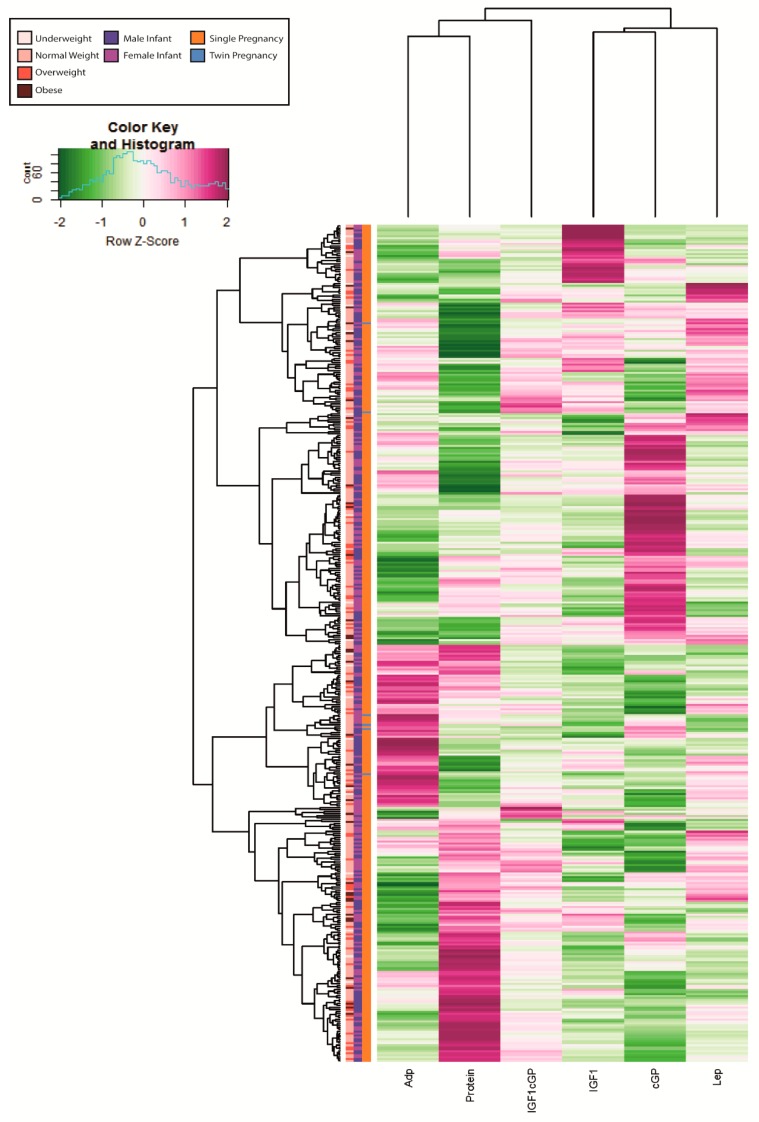
Heatmap displaying hormonal composition (z-scores) for 423 samples in the Finnish STEPS cohort. Only samples with no missing information were included in the heatmap. Adp = Adiponectin, IGF1 = Insulin-like growth factor 1, cGP = cyclic-Glycine-Proline, IGF1cGP = IGF1/cGP ratio, Lep = leptin. The missing samples have been excluded due to missing information for one or more hormone measurements.

Time of HM collection (i.e., month post-partum) in the present cohort (for leptin β = −0.030 and *p* = 0.505, for adiponectin β = −0.019 and *p* = 0.667, for IGF-1 β = 0.059 and *p* = 0.191, for cGP β = 0.033 and *p* = 0.498) did not affect any of the analytes, and thus, was not included in any further analyses. Values of the five analytes across the study population are reported in Table 2. IGF-1 and adiponectin concentrations displayed correlations with leptin (β = 0.189 for IGF-1 and β = 0.089 for adiponectin). A visualization of the general hormonal composition and clustering is shown in Figure 1.

### 3.3. Maternal Factors and HM Composition

HM leptin concentration showed a strong association with maternal pre-pregnancy weight (Table 3) and BMI (*p* < 0.001, Table 4), with overweight and obese mothers having significantly higher HM leptin concentrations. Furthermore, HM IGF-1 displayed a positive correlation with maternal weight gain during pregnancy (Table 3), which was maintained when correcting for maternal pre-pregnancy weight (*p* = 0.009, β = 0.127). Finally, the concentration of total protein in the samples was associated with maternal basic education (*p* = 0.008, Table 4). No significant association was found between maternal factors and either cGP or adiponectin.

### 3.4. Infant Factors and HM Composition

As shown in Table 3, adiponectin was positively correlated with infant birthweight (β = −0.106, *p* = 0.02). Giving birth to twins also was associated to HM adiponectin concentrations with mothers of twins secreting higher adiponectin in their milk (Table 4) as compared to mothers of singletons. As illustrated in Table 3, the total protein content of HM was correlated with infant nutritional practices and showed significant differences in association with birth mode (*p* = 0.003). The IGF-1/cGP ratio displayed a positive correlation with gestational age (β = 0.103, *p* = 0.033).

### 3.5. Maternal-Infant Interactions, Sex-Specificity and HM Composition

**Figure 2:**
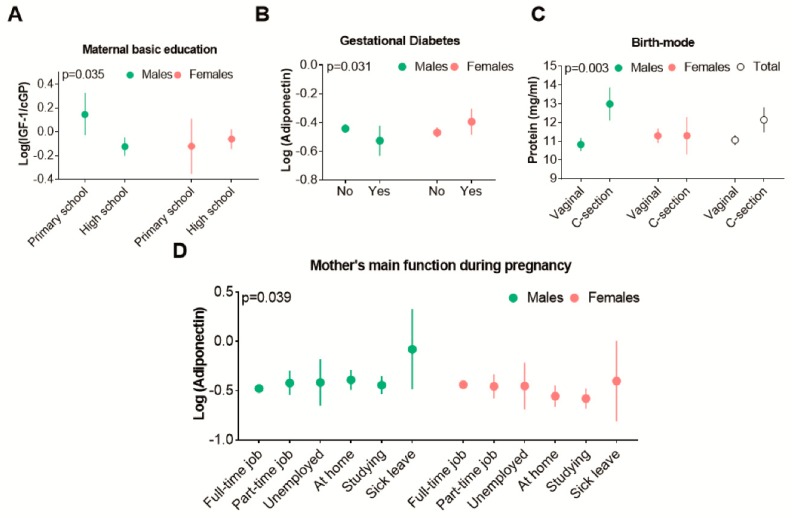
Sex-specific interactions and human milk (HM) composition. *p*-values are relative to the interaction between the two factors and HM composition (i.e., infant sex, birth-mode and milk protein). All the graphs show that maternal factors ((**A**) basic education, (**B**) gestational diabetes, (**C**) birth-mode and (**D**) mother’s occupation during pregnancy) is associated with the concentration of HM components in a sex-specific manner. Graphs represent Mean and CIs. Mothers in the Sick Leave group were only *n* = 3 (two males and one female). IGF1 = Insulin-like growth factor 1, cGP = cyclic-Glycine-Proline, IGF1cGP = IGF1/cGP ratio.

Our cohort presented evidence of sex-specific effects on HM hormonal concentrations. IGF-1/cGP ratio showed a sex-specific interaction with maternal basic education (*p* = 0.035, Figure 2A). Additionally, adiponectin concentrations were observed to be different for male and female infants in relation to GDM (*p* = 0.031, Figure 2B) and maternal work status during pregnancy (*p* = 0.039, Figure 2D). However, in the case of GDM, the sex-specific differences became non-significant when correcting the analysis for exclusive duration of breastfeeding (*p* = 0.05). Finally, total protein concentrations were elevated in mothers of males who underwent C-section (Figure 2C).

## 4. Discussion

Our findings showed an association between pre-pregnancy BMI, pregnancy factors (pregnancy weight gain, GDM, multiple birth, birth-mode and infant gestational age) and infant characteristics at birth (infant sex and birth weight) and the concentration of bioactive compounds in HM collected three months after birth. In the present study, we also report, for the first time to our knowledge, the presence and concentrations of cGP in HM.

The contribution of maternal BMI to HM adipokines [35], particularly leptin [36], is well documented and was further confirmed by our findings. Overweight and obese mothers had significantly elevated HM leptin concentrations compared to normal weight mothers. Additionally, HM IGF-1 concentrations at the time of collection was proportional to pregnancy weight-gain. Higher pregnancy weight gain could have arisen due to higher circulating IGF-1 that would have stimulated foetal growth [37], but HM IGF-1 did not correlate to infant birthweight in our cohort despite previous studies reporting increased HM IGF-1 concentrations with GDM and macrosomia [38]. Differences in dietary intakes, and thus pregnancy weight gain, might have caused the observed increases in HM IGF-1 concentration [39], although in the absence of maternal dietary information during pregnancy we cannot explore such associations. In our cohort, we observed no correlation between time of collection (i.e., month post-partum) and HM composition. This was most likely due to the fact that the period of HM collection in the present cohort spanned predominantly between 2 and 3 months post-partum (90% of samples), when HM composition is mature and the least affected by changes in lactation stage.

HM adiponectin was higher in mothers who delivered infants small for gestational age, as reported in previous studies [36] and in mothers of twins. Although the latter group was of low sample size in our cohort, the literature reports higher circulating adiponectin during twin pregnancies [40]. Elevated concentrations of circulating adiponectin are attributed to the larger placenta carried by mothers of twins [41] but are known to reduce significantly shortly after birth [40]. Therefore, it is not clear how higher concentrations of adiponectin would remain in HM of mothers who gave birth to twins three months prior to HM sample collection.

We observed that GDM mothers of males had the lowest concentration of HM adiponectin compared to GDM mothers of females and non GDM mothers of males and females, suggesting that infant sex can have a major effect on milk composition. The existing literature reports evidence that lower circulating adiponectin concentrations during pregnancy is associated with the incidence of GDM [42]. Circulating concentrations of adiponectin are also known to be sexually dimorphic, as increasing concentrations of testosterone inhibit the production of adiponectin by adipocytes [43,44]. Notably, an increase in testosterone concentrations commonly occurs in mothers who are carrying a male foetus [45]. Although in our cohort these sex-specific correlations became statistically non-significant when corrected for exclusive breastfeeding duration, the *p*-value (0.05) still suggests an interesting associative trend. Considering that the number of GDM mothers in our cohort was limited to 44 and only constituted less than 10% of the study population, further studies with a larger GDM group should investigate this association and the impact of low adiponectin intakes for male babies, since low adiponectin is a risk factor for a range of metabolic and cardiovascular disorders [46,47].

Birth-mode also displayed a sex-specific effect on HM composition, as mothers of males who underwent C-section displayed the highest amount of protein in their milk compared to all other groups. In our cohort, mothers who underwent C-section generally displayed higher protein irrespective of infant sex. However, the association between C-section and mature HM composition appears to be a novel finding in the present study. As elevated protein concentrations during early life are known to increase the risk of childhood obesity [48], it is crucial to understand whether HM protein content is altered by C-section, or whether the elevated protein content of HM constitutes an adaptation to provide more nutrients to those infants with higher requirements. In our cohort, there were no elective C-sections, and most of them were performed on mothers that were past 40 weeks of gestation (*n* = 39) compared to mothers who were less than 37 weeks of gestation (*n* = 11) and mothers who were between 37 and 40 weeks of gestation (*n* = 12). Only four of the infants born by C-section in our cohort were classified as small for gestational age, compared to 55 infants that were classified as appropriate for gestational age and two that were classified as large for gestational age. Taken together these observations, although of low sample size, may suggest that rather than constituting an adaptation to provide small babies with higher protein, the elevated concentrations of protein in HM of mothers who underwent C-section may be the result of altered metabolic pathways and therefore warrants further investigation.

Of interest, our analysis also revealed sex-specific effects linked to socioeconomic and stress-related factors. As an example, higher concentrations of adiponectin and the IGF-1/cGP ratio were observed in male infants of mothers that experienced what would be classed as more stressful pregnancies (i.e., sickness during pregnancy) or were of lower socioeconomic status (i.e., mother only completed primary school) although the size of some subgroups in these cases was extremely limited as discussed below. We also found that the amount of protein in HM was higher for mothers with a lower education background. While these observations are purely associative, we have previously reported that social influences, including maternal educational status, impact on HM stress hormone levels [30]. Given the known link between stress hormones and changes in IGF-1 and adiponectin [49], changes in stress markers may have mediated some of the associations observed. Although due to sample size limitations, cortisol and cortisone were unable to be measured in the current cohort.

The main strength of the present study is the large sample size, although the size of certain subgroups was limited due to the fact that the study was designed retrospectively. The lack of multiple HM collection time points from individual mothers prevented the analysis of HM compositional changes within subjects. Additionally, our samples were only representative of morning HM rather than the gold standard 24-h collection and considerable time passed between sample collection (2008–2010) and the biochemical analyses (2018) presented in this study, due to the retrospective nature of the present study design. However, the compounds analysed in the present study are known to be stable at −80 °C. Furthermore, because the HM samples were collected exclusively in the morning, collection timing and procedure was relatively standardised across all mothers. Many studies previously analysed HM composition around 3 months after birth. At this time, HM composition is more stable and thus less variable, resulting in comparable samples.

Matched plasma samples and access to more detailed dietary information during pregnancy and lactation would have been useful to further delineate some of the associations detailed. Although the design of the current study does not allow any detailed mechanistic insight, previous observations in a bovine model have reported on differential programming of milk production as a function of foetal sex [50]. The study by Hinde et al. showed a marked and sustained effect of mammary function that arises as a result of foetal sex, with the female sex of the foetus enhancing milk production for multiple consequential lactations.

## 5. Conclusions

Overall, our findings show that maternal-infant factors, specifically maternal pregnancy and pre-pregnancy characteristics and infant sex, interact and impact upon HM compositional profiles up to three months after birth, suggesting that HM composition may be delineated during pregnancy and may have a characteristic fingerprint for every mother-baby dyad. Such a fingerprint might be used to draw maps that would serve to characterise HM composition in association with specific pathophysiological conditions. Whether the intake of HM and hormonal concentrations associated with these pathophysiological conditions impacts on postnatal outcomes and growth trajectories on the long term remains to date unknown and should be investigated in future research. This will allow a better understanding of the role played by specific nutritional and hormonal exposures during early life, in order to implement nutrition for infants who have no access to HM but also to design strategies that would help improve possibly altered HM composition due to pathological conditions. Although we had no means to investigate “cause-effect” relationships between maternal circulating hormones, dietary intakes and bioactives in HM, in the present study, the associations we observed suggest that prospective studies designed to untangle such associations and establish key determinants (and interactions therein) related to HM composition and infant outcomes are warranted.

## Figures and Tables

**Table 1:** Overall characteristics of the study population for *n* = 501 mothers and *n* = 507 infants taking part in the Finnish STEPS cohort.

**Maternal, Pregnancy and Birth Characteristics**	*n* (%)
**Maternal age at birth, Mean (SD)**	31.1 (4.2)
**Maternal Basic Education**	
Primary school	77 (15.4)
High School	420 (84)
Other	3 (0.6)
Missing	1
**Maternal pre-pregnancy BMI (classes) and weight gain**	
Underweight	22 (4.3)
Normal weight	343 (67.7)
Overweight	91 (17.9)
Obese	51 (10.1)
Weight gain during pregnancy, Mean (SD)	12.5 (5.80)
**Maternal gestational diabetes mellitus (GDM)**	
No	460 (91.3)
Yes	44 (8.7)
Missing	3
**Mother’s main function during pregnancy**	
Work full-time	343 (68.6)
Work part-time	34 (6.8)
Unemployed	10 (2.0)
Housewife/at home	53 (10.6)
Studying	53 (10.6)
Sick leave	3 (0.6)
Other	4 (0.8)
Missing	1
**Single/multiple pregnancies**	
Singletons	495 (97.6)
Same-sex twins	5 (1)
Different-sex twins	1 (0.2)
**Birth mode**	
Vaginal	443 (87.9)
C-section	61 (12.1)
**Infant characteristics**	
**Infant sex**	
Male	273 (53.8)
Female	234 (46.2)
**Gestational age (GA)**	
>36.99 weeks GA	477 (95.2)
<37 weeks GA	21 (4.2)
Small for GA (SGA)	16 (3.2)
Appropriate for GA (AGA)	478 (94.8)
Large for GA (LGA)	10 (2)
Missing	3(0.6)
Infant birthweight, g, Mean (SD)	3489.97 ± 505.54
Infant birthweight, z-score, Mean (SD)	0.18 ± 0.889
Infant birth length, cm, Mean (SD)	50.78 ± 2.19
**Nutritional practices**	Median (Min,Max)
Duration of exclusive breastfeeding (months)	3.2 (0.0,6.5)
Duration of partial breastfeeding (months)	6.4 (0.0,38.3)
Duration of total breastfeeding (months)	9.9 (0.0,40.3)
Introduction of solid food (months postpartum)	4.0 (1.0,6.5)

The data are presented as means and standard deviation (SD) for continuous variables and as numbers (percentage) for categorical variables.

**Table 2:** Characteristics of milk samples for *n* = 501 mothers and *n* = 507 infants taking part in the Finnish STEPS cohort.

Human Milk (HM) Characteristics	Mean (SD)
Leptin (ng/mg)	0.116 (0.112)
Adiponectin (ng/mg)	0.404 (0.237)
cGP (ng/mg)	0.278 (0.222)
IGF-1(ng/mg)	0.180 (0.099)
Protein (mg/mL)	11.24 (2.147)
Lactation stage at milk sample collection (months postpartum)	2.6 (0.40)

Raw values corrected per mg of protein per ml; IGF-1, Insulin-like growth factor 1; cGP, cyclic Glycine-Proline.

**Table 3:** Simple linear regression between maternal-infant characteristics and HM adiponectin, leptin, IGF-1, cGP and total protein.

	Adiponectin (ng/mg)		Leptin (ng/mg)		IGF-1 (ng/mg)		cGP (ng/mg)		Protein (mg/mL)	
	Β	*p*	Β	*p*	β	*p*	β	*p*	β	*p*
**Maternal characteristics**										
Maternal Age (years)	0.049	0.28	−0.032	0.478	−0.074	0.10	−0.051	0.29	0.026	0.54
Maternal pre-pregnancy height (cm)	0.042	0.36	−0.035	0.44	−0.008	0.86	−0.068	0.17	0.018	0.69
Maternal pre-pregnancy weight (kg)	0.045	0.32	0.159	<0.001 **	−0.023	0.61	−0.066	0.18	0.050	0.27
Maternal pregnancy weight-gain (kg)	−0.079	0.10	−0.015	0.76	0.104	0.03 *	−0.028	0.59	0.012	0.80
**Infant characteristics**										
Gestational age (weeks)	−0.054	0.23	0.065	0.15	0.028	0.54	0.063	0.20	0.026	0.56
Birthweight (g)	−0.106	0.02 *	0.039	0.39	−0.025	0.58	0.058	0.23	0.021	0.63
Birth-length (cm)	−0.043	0.35	0.001	0.99	−0.006	0.89	−0.017	0.73	−0.007	0.87
**Nutritional practices**										
Total breastfeeding duration (months)	−0.137	0.01 **	−0.042	0.41	−0.013	0.80	−0.035	0.53	−0.135	0.01 **
Exclusive breastfeeding duration (months)	−0.058	0.23	0.039	0.42	0.028	0.56	0.057	0.27	−0.146	0.002 **
Partial breastfeeding duration (months)	−0.088	0.1	−0.014	0.80	−0.035	0.51	−0.063	0.28	−0.062	0.24
Introduction of solid foods (months)	−0.081	0.09	−0.032	0.50	0.004	0.93	0.034	0.52	−0.101	0.04 *

β stands for beta coefficient of the regression and *p* for *p*-value; * *p* < 0.05, ** *p* < 0.01, All hormones were log-transformed; IGF-1, Insulin-like growth factor 1; cGP, cyclic Glycine-Proline.

**Table 4:** Associations between maternal-infant characteristics and HM adiponectin, leptin, IGF-1, cGP and protein according to MANOVA analyses.

	Adiponectin (ng/mg)		Leptin (ng/mg)		IGF-1 (ng/mg)		cGP (ng/mg)		Protein (mg/mL)	
	Mean Difference (95%CI)	p^a^	Mean Difference (95%CI)	p^a^	Mean Difference (95%CI)	p^a^	Mean Difference (95%CI)	p^a^	Mean Difference (95%CI)	p^a^
**Maternal-infant characteristics**										
**BMI**										
Underweight	−0.022 (−0.174, 0.129)	1	−0.003 (−0.133, 0.127)	1.000	−0.027 (−0.150, 0.096)	1	1.894 (−34.541, 38.328)	1	−0.225 (−1.797, 1.347)	1
Normal weight	Reference		Reference		Reference		Reference		Reference	
Overweight	0.025 (−0.061, 0.110)	1	−0.085 (−0.159, −0.013)	**0.013**	−0.056 (−0.014, 0.125)	0.202	5.757 (−14.844, 26.358)	1	0.017 (−0.872, 0.906)	1
Obese	0.011 (−0.108, 0.130)	1	−0.137 (−0.238, −0.035)	**0.003**	0.011 (−0.086, 0.107)	1	4.755 (−23.786, 33.296)	1	−0.724 (−1.955, 0.507)	0.717
**Gestational Diabetes**										
No	Reference		Reference		Reference		Reference		Reference	
Yes	−0.012 (−0.099, 0.074)	0.779	−0.018 (−0.093,0.058)	0.646	0.021 (−0.048, 0.091)	0.55	3.962 (−16.862, 24,787)	0.708	0.120 (−0.785, 1.024)	0.795
**Basic Education**										
Primary school	−0.008 (−0.026, 0.075)	1	−0.009 (−0.084, 0.066)	1	0.047 (−0.022, 0.116)	0.311	2.061 (−18.301, 22.424)	1	−1.103 (−1.974, −0.232)	**0.008**
High school	Reference		Reference		Reference		Reference		Reference	
Other	0.191 (−0.100, 0.481)	0.346	0.160 (−0.101, 0.422)	0.421	−0.83 (−0.323, 0.157)	1	2.816 (−68.050, 73.682)	1	0.677 (−2.354, 3.709)	1
**Main function during pregnancy**										
Work full-time	Reference		Reference		Reference		Reference		Reference	
Work part-time	−0.019 (−0.161, 0.124)	1	0.070 (−0.059, 0.198)	1	0.016 (−0.102, 0.133)	1	7.244 (−27.957, 42.446)	1	−0.099 (−1.598, 1.399)	1
Unemployed	−0.024 (−0.287, 0.239)	1	−0.040 (−0.278, 0.198)	1	0.099 (−0.119, 0.316)	1	4.713 (−60.515, 69.942)	1	−1.536 (−4.313, 1.241)	1
Housewife/at home	0.015 (−0.108, 0.138)	1	−0.013 (−0.125, 0.098)	1	0.025 (−0.077, 0.127)	1	5.141 (−25.457, 35.740)	1	−0.255 (−1.528, 1.078)	1
Studying	0.054 (−0.060, 0.169)	1	−0.002 (−0.102, 0.105)	1	−0.035 (−0.130, 0.060)	1	6.329 (−22.075, 34.732)	1	0.051 (−1.159, 1.260)	1
Sick leave/sickness pension	−0.217 (−0.667, 0.233)	1	0.342 (−0.065, 0.748)	0.222	0.169 (−0.204, 0.542)	1	8.235 (−157.434, 119.904)	1	−4.269 (−9.024, 0.485)	0.132
Other	0.121 (−0.514, 0.757)	1	−0.002 (−0.576, 0.572)	1	−0.365 (−0.891, 0.162)	0.727	−0.034 (−157.639, 157.571)	1	−1.310 (−8.020, 5.400)	1
**Depression**										
No	Reference		Reference		Reference		Reference		Reference	
Yes	0.119 (−0.030, 0.268)	0.118	−0.060 (−0.195, 0.074)	0.38	−0.003 (−0.127, 0.120)	0.956	0.898 (−35.545, 37.342)	0.961	−1.158 (−2.737, 0.422)	0.15
**Birth mode**										
Vaginal	Reference		Reference		Reference		Reference		Reference	
C-section	−0.026 (−0.096, 0.043)	0.451	0.049 (0.0011, 0.109)	0.111	0.047 (−0.009, 0.102)	0.099	2.255 (−14.386, 18.897)	0.79	−1.083 (−1.786, −0.380)	**0.003**
**Twins**										
No	Reference		Reference		Reference		Reference		Reference	
Yes	−0.163 (−0.286, −0.041)	**0.009**	0.081 (−0.030, 0.191)	0.152	0.054 (−0.049, 0.156)	0.303	2.585 (−27.438, 32.609)	0.866	1.037 (−0.268, 2.341)	0.119
**Infant sex**										
Male	Reference		Reference		Reference		Reference		Reference	
Female	0.013 (−0.033, 0.058)	0.588	0.014 (−0.027, 0.055)	0.497	0.005 (−0.032, 0.043)	0.775	−5.743 (−16.734, 5.249)	0.305	−0.171 (−0.651, 0.308)	0.483

Values are based on logarithmic transformed concentrations (log10), with the exception of protein. All Mean differences are based on estimated marginal means. All hormones were included as response variables in every model. Single factors were included as independent variables in every model. The sex of the infant was inserted as a co-factor in each model to explore sex-specific differences and interactions; ^a^ adjustment for multiple comparisons: Bonferroni; IGF-1, Insulin-like growth factor 1; cGP, cyclic Glycine-Proline.
